# Reproducibility of corneal, macular and retinal nerve fiber layer thickness measurements using the iVue-100 optical coherence tomography

**DOI:** 10.4314/ahs.v17i4.33

**Published:** 2017-12

**Authors:** Khathutshelo P Mashige

**Affiliations:** University of KwaZulu-Natal, Discipline of Optometry

**Keywords:** Corneal thickness, macular thickness, retinal nerve fiber layer thickness, iVue-100, reproducibility

## Abstract

**Purpose:**

To determine the intra-session and inter-session reproducibility of corneal, macular and retinal nerve fiber layer thickness (RNFL) measurements with the iVue-100 optical coherence tomography in normal eyes.

**Methods:**

These parameters were measured in the right eyes of 50 healthy participants with normal vision. Six scans each for corneal thickness, macular and optic nerve head were taken on one day (intra-session), followed by similar repeated measures on five separate days (inter-session). Reproducibility was computed using intra-class correlation coefficient (ICC), coefficient of variation (COV), and test-retest variability (TRV).

**Results:**

For intra-session reproducibility, the ICC, COV and TRV values for mean corneal thickness were 0.924, 2.82%, and 3.06 µm respectively. For the mean macular thickness, they were 0.978, 4.64% and 4.51 µm respectively, while for mean RNFL thickness they were 0.946, 3.19%, and 5.66 µm respectively. Inter-session values for mean corneal thickness were 0.926, 2.65% and 3.48 µm, and 0.916, 2.24% and 2.03 µm for mean macular thickness. For mean RNFL thickness, they were 0.962, 2.21%, and 4.72 µm respectively.

**Conclusion:**

There was good reproducibility of all measured parameters. However, mean RNFL thickness measurements were the most reproducible, suggesting that this may be the best parameter to use to determine measured changes over time.

## Introduction

Corneal thickness measurements are important for the planning of refractive surgery, diagnosis of glaucoma, and for therapeutic applications such as monitoring corneal oedema and endothelial function.^1^ Accurate and reliable determination of macular and retinal nerve fiber layer (RNFL) thicknesses have become increasingly necessary in the diagnosis and management of macular and optic nerve head anomalies, respectively.^1^

The introduction of optical coherence tomography (OCT) has been a major development in high resolution imaging of ocular tissues to improve our ability to diagnose and manage various ophthalmic diseases. The iVue-100 and RTVue-100 (Optovue, Inc, Fremont, CA) are relatively new-generations of spectral domain optical coherence tomography (SD-OCT) devices that image the anterior and posterior segments with faster acquisition and better image quality compared with previous generations of this technology such as time-domain optical coherence tomography (TD-OCT).^2^ Studies have shown that the RTVue-100 yielded excellent reproducibility for corneal thickness and RNFL measurements.^3^,^4^ The shortterm repeatability and the correlation between supine and sitting measurements of optic nerve head (ONH) and RNFL parameters obtained with the iVue SD-OCT in healthy participants and glaucoma patients have been evaluated in a previous study.^5^ The results showed that the ability of the iVue SD-OCT to quantify ONH and RNFL parameters ranged from good to excellent in both body positions and comparable to reported values for stationary SD-OCT instruments in the sitting position. However, the intra-session and inter-session reproducibility of this device for corneal and macular parameters in any body position has not been assessed. In addition, values obtained by RTVue-100 and iVue-100 machines may not be inter-changeable. The iVue is a portable device that enables imaging in different body positions and can therefore be used on domiciliary visits as well as outside the limits of a consulting room.^5^

Reproducibility of measurements is an essential quality in determining the utility of a device used for clinical and research purposes.^2^ The aim of this study was therefore to determine the reproducibility of corneal, macular and RNFL thickness measurements in normal eyes using the iVue-100 SD-OCT.

## Subjects and methods

The study was approved by the University of KwaZulu-Natal's Biomedical Research and Ethics Committee and adhered to the tenets of the Declaration of Helsinki. All participants gave written informed consent after the nature of the study had been explained to them. Participants were assured that their data would be presented anonymously to protect their identity. The study was conducted at the Discipline of Optometry, School of Health Science, University of KwaZulu-Natal, and a convenience sampling method was used to recruit 50 healthy participants from the employee pool and student population of the institution.

All participants underwent a complete ophthalmic examination that included a review of ocular and medical histories, best-corrected visual acuity measurements, slit-lamp examination, intraocular pressure and corneal thickness measurements with the Nidek NT530P (Tonopachy™, Nidek, Japan), and a fundus examination. Only individuals who had normal eyes (defined as an absence of any corneal and retinal diseases, having had no history of eye diseases, a best-corrected visual acuity of 20/25 or better, a spherical refraction within ± 4.0 diopters, and a cylindrical refraction within ± 3.0 diopters) were included. The exclusion criteria consisted of a history of any eye surgery, clinical evidence of corneal, optic nerve or retinal pathology, a cup-to-disc ratio of > 0.5, and an intraocular pressure of > 21 mm Hg. Contact lens wearers were also excluded from the study.

All OCT measurements were taken between 2 and 5 pm in all sessions, with all subjects having been awake for at least two hours before the measurements were taken. A trained and experienced operator performed all the OCT examinations according to the analysis protocol and variables. The iVue-100 OCT device was fixed to a table in front of which each participant sat to undergo the OCT scans (corneal, macular and optic nerve head) on the same eye on the first day and five separate sessions on different days. The same device was used by the same operator for all the sessions. Each participant was requested to maintain proper fixation to render the readings reliable. The joystick was used to move the scanner head until the pupil was in the middle of the reticule on the video screen and the iris was in focus. The image displayed automatically when proper alignment and focus were achieved. The ocular (front objective) lens of the iVue-100 was cleaned using lens paper with lens cleaning solution between each participant. The chin rest and forehead rest were cleaned with a towel dipped in disinfecting solution. All scans were obtained in dim illumination conditions to allow for physiological dilation of the pupil. All print outs were obtained after completion of the study.

Images of the ocular microstructures are provided by a scanning laser diode to emit a scan beam with a mean wavelength of 840 nm and a standard deviation of 10 nm.^2^ The iVue-100 OCT provides a fast scanning of the tissue (26,000 A-scans per second), achieving an axial length resolution of 5 µm.^2^ The corneal thickness (taken with a CAM lens attached), macular and the optic nerve head (ONH) iVue-100 (Optovue, Inc.) protocols were used to obtain corneal, macular and RNFL imaging and parameters respectively.

The full 6 mm diameter corneal thickness mapping with minimum thickness indicator was scanned. The retinal map scan protocol consists of a raster pattern of 13 horizontal (6 mm) line scans of 512 A-scans and 7 horizontal (6 mm) line scans of 1024 A-scans within the central 1.5 mm vertical zone.^2^,^6^ The output from the pachymetry scans was the mean corneal thickness, as well as the average corneal thickness in the superior, nasal, inferior and temporal quadrants. The ONH scan measures RNFL thickness along a circle 3.45 mm in diameter centred at the optic disc using a map created by a combination of information from six circles and 12 lines.^2^,^6^ The macular and ONH scans determine the average macular and RNFL thicknesses as well as the temporal, superior, nasal and inferior average macular and RNFL thicknesses.

Following each examination, the images were deemed acceptable if the cornea or retina were clearly visible and distinguishable in every scan, no eye movement or blinking artefacts occurred during the examination, and the full depth and extent of the cornea or retina was visualized in each scan image. The quality of the image was displayed after each scan and was based on the intensity of the reflected light. This is described as the signal strength index (SSI), and ranges from near 0 (no signal) to approximately 90 (very strong signal). Only the scans with a SSI of 45 and higher were used for analysis as per the manufacturer's recommendations.^6^

To measure intra-session variability, six sets each of corneal, macular and RNFL scans were taken in quick succession in one day. The same scans were taken on the same participants by the same operator on 5 different days, with each session separated by 5 days, without using the repeat scan function on the iVue-100 device. The six sets of each parameter taken on the first day were analyzed for intra-session variability and the average of 6 scans for each parameter on each day was used for inter-session variability.

The number of measurements per subject, the lower confidence interval (CI) and the intra-class correlation coefficient (ICC) were used to determine the desired sample size. Giraundeau and Mary,^7^ as well as Fleiss,^8^ suggested that the lower ICC cut-off for any good reproducibility study is 0.75. If five measurements per subject are used, 40 subjects would be needed to yield an ICC of 0.8 with a lower CI of 0.73. In this study, six measurements per subject were taken, and therefore 50 subjects were considered to be a reasonable sample size.

The reproducibility of iVue-100 measurements was assessed using the intra-class correlation coefficient (ICC), the coefficient of variation (COV), and the test-retest variability (TRV). The values were determined for each of the corneal [mean, and 4 quadrants (temporal, superior, nasal, and inferior)], macular [overall average, 4 quadrants (temporal, superior, nasal, and inferior)], and RNFL [i.e. overall global, 4 quadrants (temporal, superior, nasal, and inferior)] parameters. Lower and upper 95% CI of ICC were determined for each participant. Intra-class correlation coefficients (ICCs) were determined using a two-way random effects analysis of variance (ANOVA). “The ICC represents the ratio of the between-cluster variance. It is thus an indicator of the proportion of variability attributable to the measurement itself, as opposed to the variation between different individuals in the parameter being measured.”^9^ In addition, “values of ICCs range from 0 to 1, where ‘0’ indicates perfect disagreement and ‘1’ indicates perfect agreement between repeated measurements.”^10^

Coefficient of variation (COV), expressed in percentage, was computed as the standard deviation of variability divided by mean thickness separately for the intra-session and inter-session parameters. Test-retest variability (measured in micrometres) was determined as twice the square root of the variance among repeated measures for each of the variables assessed. Each of these were calculated as both inter-session and intra-session parameters. All statistical analyses were conducted with SPSS software (version 19, SPSS, Chicago, IL, USA) and the results with p < 0.05 were interpreted as statistically significant.

## Results

Fifty right eyes of 50 normal participants with a mean age of 26.6 ± 4.8 years (range, 19 to 38 years) were studied and analyzed. There were 25 women and 25 men, and 39 of whom were Black and 11 were Indians. For each participant six sets of results were obtained for each parameter; the parameters being the corneal, macular and RNFL thickness measurements in each quadrant. The OCT was well tolerated by all participants and their scans were good, with SSI values of above 70.

For intra-session reproducibility, the ICC values for corneal thickness measurements were 0.8 or higher and COV was under 7% for all quadrants. The mean corneal thickness showed the least variability compared to other corneal quadrants (Table 1). For the macular and RNFL thickness measurements in all four quadrants, the ICC values were 0.9 or higher and COV was under 7%. Intra-session reproducibility values were best in the nasal macular quadrant compared to other macular quadrants and worst in the temporal RNFL quadrant compared to other RNFL quadrants (Table 1). Overall, compared to the macular and RNFL values, the corneal thickness values showed the worst intra-session reproducibility as shown in Table 1.

**Table 1:** Intra-session reproducibility

	Intra-class correlation coefficient (95% CI)			COV (%)			Test-retest variability (µm)		

Quadrant	Cornea	Macular	RNFL	Cornea	Macular	RNFL	Cornea	Macular	RNFL
Average	0.898(0.887–0.911)	0.934(0.928–0.950)	0.946(0.938–0.952)	2.51	2.39	2.11	5.06	4.86	4.48
Temporal	0.887(0.861–0.893)	0.922(0.914–0.929)	0.907(0.899–0.914)	5.29	5.44	5.22	13.92	13.67	13.22
Superior	0.896(0.884–0.908)	0.938(0.926–0.947)	0.932(0.924–0.943)	3.56	4.77	3.46	6.88	6.37	6.16
Nasal	0.892(0.880–0.902)	0.944(0.934–0.951)	0.928(0.920–0.931)	6.30	6.23	6.44	8.76	8.51	8.37
Inferior	0.894(0.890–0.904)	0.926(0.911–0.935)	0.930(0.921–0.939)	3.98	3.67	3.38	9.90	9.64	9.07

CI: Confidence interval, COV: Coefficient of variation, RNFL: Retinal nerve fiber layer

Similar to the intra-session measurements, inter-session ICC values for corneal thickness measurements were 0.8 or higher and COV was under 7% for all quadrants (Table 2). Similarly, the macular and RNFL were 0.9 or higher and under 7% for ICC and COV respectively in all quadrants. Table 2 shows that average ICC for cornea, macular and RNFL thicknesses were 0.898, 0.931 and 0.943 respectively, suggesting that RNFL measurements had the best inter-session measurement agreements and the cornea had the worst.

**Table 2:** Inter-session reproducibility

	Inter-class correlation coefficient (95% CI)			COV (%)			Test-retest variability (µm)		

Quadrant	Cornea	Macular	RNFL	Cornea	Macular	RNFL	Cornea	Macular	RNFL
Average	0.898(0.889–0.905)	0.931(0.923–0.941)	0.943(0.930–0.947)	2.62	2.46	2.33	5.13	4.69	4.97
Temporal	0.887(0.876–0.893)	0.926(0.919–0.933)	0.901(0.888–0.909)	5.66	5.59	5.28	14.04	13.83	13.59
Superior	0.891(0.879–0.900)	0.933(0.924–0.943)	0.930(0.922–0.935)	3.96	4.92	3.89	6.97	6.56	6.81
Nasal	0.895(0.882–0.897)	0.941(0.930–0.946)	0.937(0.931–0.943)	6.49	6.66	6.63	8.92	8.79	8.85
Inferior	0.896(0.888–0.901)	0.928(0.918–0.939)	0.933(0.928–0.939)	4.23	3.80	3.46	9.98	9.85	9.88

CI: Confidence in terval, COV: Coefficient of variation, RNFL: Retinal n erve fiber layer

## Discussion

The iVue-100 is a relatively new generation SD-OCT device used to qualitatively and quantitatively assess ocular structures important to the pathogenesis and anatomical variations of several ocular diseases, and to identify approaches to successful treatment.^11^ Assessing the reproducibility of an instrument used for ophthalmic examination is important, as it affects the diagnostic accuracy and the ability to detect changes over time.^12^ Consequently, the goal of this study was to assess the reproducibility of the iVue-100 OCT corneal, macular and RNFL thickness measurements. To the best of the author's knowledge, this is the first report on the reproducibility of these parameters using the iVue-100 OCT in normal subjects. The current study demonstrates that iVue-100 OCT corneal, macular, and RNFL thickness measurements are reproducible.

The ICC values for corneal, macular, and RNFL thickness measurements in all quadrants assessed were above 0.75, a generally accepted lower value of good reproducibility. 7,8 Similarly, the COV values in all quadrants were less than 10%, indicating good reproducibility of the iVue-100 OCT for all the parameters assessed. A device with a COV of < 10% is regarded as having high reproducibility and a COV < 5% indicates very high reproducibilty.^13^

The corneal measures were lower (showed least reproducibility) in comparison to ONH or macular measures. This could be due to factors such as higher variations in corneal thickness compared to retina, or the presence of the choroid (a more stable tissue) behind the retina versus aqueous (a more variable tissue) behind the cornea. Although corneal thickness measurements were least reproducible compared to the other parameters assessed, their ICC and COV values were still higher than 0.8 and less than 10% respectively (Tables 1 and 2). Previous studies on reproducibility of corneal thickness measurements using other commercially available OCTs (e.g. Visante, 3D CAS-OCT, Slit-lamp OCT, RTVue-100) have shown ICC values ranging from 0.767–0.999, and COVs of less than 3.7%.^14^,^15^ For example, Ishibazawa et al^15^ reported that the RTVue had the best reproducibility with a mean ICC for corneal thickness of 0.980 and a mean COV of 3.64%. This could be due to the fact that the RTVue OCT obtains a high resolution of the cornea with similar shorter operating wave length of 840 nm compared to other devices, such as the Visante, which have longer operating wavelengths.^16^

The iVue-100 is one of the first instruments capable of automatically measuring macular thickness as the combined ganglion cell complex composed of macular RNFL, ganglion cell layer (GCL) and inner plexiform layer (IPL).^9^ In this study, reproducibility of the macular thickness measurements was good, with mean ICC and COV values of 0.934 and 2.39% respectively for intra-session variability, and 0.931 and 2.46% for inter-session variability (Tables 1 and 2). Previous studies reported ICC values of 0.9 and above, and COV values of less than 3.5%.^17^,^18^ Therefore, the results obtained in this study are similar to those reported in previous studies using different OCT devices.

Studies using several imaging devices to analyze and quantify the RNFL thickness have shown that the reproducibility of these measurements are of paramount importance in diagnosing glaucoma, as well as in monitoring its progression and therapeutic interventions.^19^ In the present study, the mean ICC, COV and TRV values were 0.946, 2.11% and 4.48 respectively for intra-session reproducibility, and 0.943, 2.33% and 4.97 respectively for inter-session reproducibility (Tables 1 and 2), indicating a high degree of RNFL measurement reproducibility with the iVue OCT device. These findings are similar to those reported by previous investigators, who showed that the reproducibility for RNFL thickness measurements are equally excellent in normal eyes for Spectralis SD,^20^ Stratus SD-OCT,^18^ Cirrus HD-OCT,^20^ Cirrus SD-OCT^21^ and RTVue.^22^ Therefore, the results of the present study are similar to those reported in previous findings with other types of OCTs.

The nasal and temporal quadrants showed higher COV values compared to the superior and inferior quadrants, however, their lower 95% CI values were still greater than 0.75, indicating good reproducibility (Table 2). Other studies with different OCT devices have also shown the nasal and temporal RNFL thickness measurements to be the least reproducible in comparison to other quadrants. 2,20 A possible reason for the higher COV seen in the nasal and temporal quadrants in this and other studies could be due to the normal retinal anatomy, in that the RNFL bundles are thicker in the superior and inferior than the nasal and temporal quadrants.^19^ Therefore, higher COV values would be obtained in the normally thinner nasal and temporal quadrants as the COV is calculated as the standard deviation divided by the mean RNFL thickness.^19^

A few limitations of this study must be acknowledged. Firstly, the minimal motion during image acquisition was due to the nature of subject selection, which included relatively young participants, with an average age of 26.6 ± 4.8 years (range, 19 to 38 years) with good vision. Further investigations regarding the reproducibility of these parameters are therefore needed in subjects of older age and those with low vision. Secondly, the study was conducted in normal eyes of healthy subjects and the variability of the iVue-100 OCT in assessing and monitoring these parameters in subjects with corneal diseases or other ocular pathologies therefore remains for future investigation.

Despite these limitations, this is the first study on the reproducibility of corneal, macular and RNFL layer thickness measurements with the iVue-100 OCT. In conclusion, in this population of young healthy adults with normal vision, there was good reproducibility of corneal, macular and RNFL thickness measurements acquired with iVue-100 SD-OCT. Knowing that these parameters are reproducible with this device adds to the improved clinical efficiency of diagnosis and follow-up of both normal corneas and retinas.
